# Enhanced angiogenesis in ischemic skeletal muscle after transplantation of cell sheets from baculovirus-transduced adipose-derived stromal cells expressing VEGF165

**DOI:** 10.1186/s13287-015-0199-6

**Published:** 2015-10-26

**Authors:** Pavel I. Makarevich, Maria A. Boldyreva, Evgeny V. Gluhanyuk, Anastasia Yu Efimenko, Konstantin V. Dergilev, Evgeny K. Shevchenko, Georgy V. Sharonov, Julia O. Gallinger, Polina A. Rodina, Stepan S. Sarkisyan, Yu-Chen Hu, Yelena V. Parfyonova

**Affiliations:** Laboratory of Angiogenesis, Russian Cardiology Research and Production Complex, Moscow, 121552 Russia; Laboratory of Regenerative Medicine, Medical Research and Educational Centre of Moscow State University, Moscow, 119192 Russia; Faculty of Medicine, M.V. Lomonosov Moscow State University, Moscow, 119192 Russia; Department of Chemical Engineering, National Tsing Hua University, Hsinchu, 300 Taiwan

## Abstract

**Introduction:**

Cell therapy using adipose-derived stromal cells (ADSC) is an intensively developing approach to promote angiogenesis and regeneration. Administration technique is crucial and among others minimal constructs - cell sheets (CS) have certain advantages. Delivery of CS allows transplantation of cells along with matrix proteins to facilitate engraftment. Cells’ therapeutic potential can be also increased by expression of proangiogenic factors by viral transduction. In this work we report on therapeutic efficacy of CS from mouse ADSC transduced to express human vascular endothelial growth factor 165 a/a isoform (VEGF165), which showed potency to restore perfusion and protect tissue in a model of limb ischemia.

**Methods:**

Mouse ADSC (mADSC) isolated from C57 male mice were expanded for CS formation (10^6^cells per CS). Constructs were transduced to express human VEGF165 by baculoviral (BV) system. CS were transplanted subcutaneously to mice with surgically induced limb ischemia and followed by laser Doppler perfusion measurements. At endpoint animals were sacrificed and skeletal muscle was evaluated for necrosis and vessel density; CS with underlying muscle was stained for apoptosis, proliferation, monocytes and blood vessels.

**Results:**

Using BV system and sodium butyrate treatment we expressed human VEGF165 in mADSC (production of VEGF165 reached ≈ 25-27 ng/ml/10^5^ cells) and optimized conditions to ensure cells’ viability after transduction. Implantation of mock-transduced CS resulted in significant improvement of limb perfusion, increased capillary density and necrosis reduction at 2 weeks post-surgery compared to untreated animals. Additional improvement of blood flow and angiogenesis was observed after transplantation of VEGF165-expressing CS indicating enhanced therapeutic potential of genetically modified constructs. Moreover, we found delivery of mADSC as CS to be superior to equivalent dose of suspended cells in terms of perfusion and angiogenesis. Histology analysis of extracted CS detected limited proliferation and approximately 10 % prevalence of apoptosis in transplanted mADSC. Significant vascularization of CS and infiltration by monocytes were found in both – BV-transduced and control CS indicating graft and host interaction after transplantation.

**Conclusions:**

Delivery of ADSC by subcutaneous transplantation of CS is effective for stimulation of angiogenesis and tissue protection in limb ischemia with a potential for efficacy improvement by BV transduction to express VEGF165.

**Electronic supplementary material:**

The online version of this article (doi:10.1186/s13287-015-0199-6) contains supplementary material, which is available to authorized users.

## Introduction

Since the initial success of cell therapy for ischemic diseases many attempts have been made to increase its efficacy. Mesenchymal stem/stromal cells including adipose-derived stromal cells (ADSC) are a promising cell source for this application. ADSC are considered an attractive candidate for therapeutic use because of their availability (from subcutaneous fat), feasible expansion, and established regenerative and angiogenic potential [1, 2].

Efficacy of cell therapy is defined by a whole spectrum of factors ranging from cell type and origin [3, 4] to passage number and expansion conditions [5]. Recently attempts have been made to enhance the therapeutic properties of the ADSC modification approach to increase secretion of growth factors and “tune up” the paracrine effects, which play a cornerstone role in their beneficial action [6, 7]. Growth factors production can be increased by a spectrum of gene-delivery tools either viral or non-viral [7]. Multiple studies and our own observations have shown that modification of ADSC does not affect their differentiation and proliferation capacity and may increase their therapeutic potential [8–10].

Choice of vector for gene delivery is a key point for successful transduction of ADSC and we focused on recombinant baculovirus (BV). BV is non-pathogenic in mammals, induces transient (approximately 30 days) production of protein [11, 12], and has minimal chance of integration into host genome [13, 14]. BV-based methods have been established for transduction of mammalian cells and optimized to achieve high expression and prolonged production period [12]. In recent years due to its properties and high transduction efficacy BV has become a tool used in a wide array of applications including vaccination [15], miRNA delivery for tumor suppression [16, 17], etc.

Previously, we developed a recombinant system with extended level and period of expression comprising two BVs carrying cDNAs of: 1) human vascular endothelial growth factor, 165 amino acid isoform (VEGF165) flanked by FRT sequences, and 2) yeast FLP recombinase. The effect of FRT/FLP relies on the generation of minicircle DNA by FRT-targeted excision and has been utilized to overexpress growth factors for ADSC-mediated bone repair [9, 18] and cartilage regeneration [10] and to enhance cells’ regenerative and pro-angiogenic potency [19].

The second issue is method of delivery to the damaged tissue, which may influence ADSC’s therapeutic efficacy. It has been shown that injection of suspended cells seems to have certain drawbacks. First, passage through the needle or catheter may disrupt the cells mechanically [20]. Another factor hindering survival of dispersed cells is anoikis, which is apoptosis induction after loss of contact with other cells [20]. Furthermore, after transplantation cells may be exposed to an inflamed hypoxic environment of damaged tissue, which results in drastic reduction of their number and, thus, therapeutic effect [21–23].

These described obstacles may putatively be circumvented by delivery of cells as minimally engineered constructs known as “cell sheets” (CS). This approach relies on administration of expanded cells (in particular ADSC) in a complex with extracellular matrix proteins they produced after seeding at high density. CS can be detached from culture dishes and transplanted to a lesion as a whole-mount patch keeping cell-to-cell junctions intact [24].

Application of CS to enhance cell survival found support in a comparative CS vs. injection study conducted in a rat model of myocardial infarction. Using a bioluminescent method the authors reported higher survival of transplanted rat neonatal cardiomyocytes after CS delivery compared to dispersed cell injection [25]. This observation was concordant with better cardiac function in animals from the CS group compared to rats treated with intramyocardial injection of cells in the same quantity.

Evaluation of ADSC-based CS in a rabbit model of myocardial infarction showed its positive effects on left ventricle geometry, ejection fraction, and vascularization [24, 25]. Still another disorder afflicting a large cohort of patients is peripheral artery disease (PAD) where cell therapy has certain promise despite clinical trials, which put its efficacy in doubt [26, 27]. CS-based methods provide an excellent opportunity to improve cell therapy for PAD and can be used during vascular grafting, reconstructive surgery, and ulcer treatment.

In this research, we established conditions for mouse ADSC (mADSC) transduction by recombinant BV. We also developed a protocol for subcutaneous transplantation of CS to mouse ischemic hind limb, which resulted in significant improvement of perfusion. CS from VEGF165-expressing mADSC showed improved therapeutic potential, which resulted in more effective restoration of blood flow. Histological studies focused on CS engraftment and induction of angiogenesis in skeletal muscle. Our data provide novel insights into approaches to enhance the efficacy of CS-based cell therapy of ischemic diseases because this study has been focusing on the combination of biomaterials and cells for angiogenesis.

## Methods

### Reagents and antibodies

#### Cell culture reagents

Dulbecco’s modified Eagle’s medium (DMEM), α-minimal essential medium (α-MEM), 100× L-glutamine, 100× antibiotic/antimycotic solution were purchased from Gibco (Gaithersburg, MD, USA); cell culture grade fetal bovine serum and 0.25 % trypsin-EDTA solution were purchased from HyClone (Logan, UT, USA); Grace’s insect medium was purchased from Sigma-Aldrich (St. Louis, MO, USA); Hanks balanced salt solution (HBSS), Dulbecco phosphate-buffered solution (PBS), Versene solution and cell culture-grade 7.5 % NaHCO_3_ solution were purchased from Paneco LTD (Moscow, Russia).

#### Histological and cell labeling dyes

Mayer’s hematoxylin solution was purchased from Dako (Glostrup, Denmark), alcoholic eosin Y solution, trypan blue 0.4 % solution and DAPI were purchased from Sigma-Aldrich; Celltracker Green CMFDA and Red CM-Dil vital dyes were purchased from Life Technologies (Carlsbad, CA, USA).

#### Antibodies

Pharmingen® rat anti mouse PECAM (CD31) monoclonal antibodies (Cat#550274) were purchased from BD Biosciences (Franklin Lakes, NJ, USA); mouse polyclonal anti α-SMA FITC-conjugated antibodies (Cat#F3777) were purchased from Sigma-Aldrich; rabbit polyclonal anti Ki-67 (Cat#ab16667) and rat monoclonal anti CD68 (Cat#ab5344) antibodies were purchased from Abcam (Cambridge, UK); rabbit polyclonal anti cleaved caspase-3 antibodies (Cat#9664S) were purchased from Cell Signaling Technology (Boston, MA, USA). Secondary fluorescent antibodies AlexaFluor®594-conjugated donkey anti rat (Cat#A21209) and AlexaFluor®594-conjugated donkey anti rabbit (Cat#A21207) were purchased from Invitrogen (Carlsbad, CA, USA);. Vectastain ABC kit (Rat IgG) (Cat#S-5000) was purchased from Vector Labs (Burlingame, CA, USA) and used for visualization of CD68 staining.

### Animal strain and ethical approval

We used nine- to ten-week-old C57/B6 male mice for ADSC isolation and hind limb ischemia model. Mice were purchased from Puschino SPF-grade breeding facility (Puschino, Russia). After acclimation, all animals received standard food and water ratios according to in-house rules of husbandry. Euthanasia was conducted after isoflurane anesthetization by secondary cervical dislocation. Surgical manipulations and euthanasia procedures were developed in compliance with National and European Union directives and were approved by the Institutional Ethics Board for Animal Care (Cardiology Research and Production Complex; permit #385.06.2009).

### Mouse ADSC isolation and expansion

Mouse adipose-derived stromal cells were obtained from subcutaneous adipose tissue of male C57/B6 mice (eight- to ten-weeks old) as previously described [8]. Isolated cells were cultured in complete (10 % FBS) 4.5 g/L D-glucose DMEM on uncoated Corning culture dishes under standard conditions at 37 °С and 5 % СО_2_. When reaching 80 % confluent monolayer mADSC were passaged at a 1:2 ratio after detachment by 0.05 % trypsin/EDTA solution. For all experimental procedures, including CS generation and preliminary testing, we used early passage (P3-P4) mADSC.

### Baculovirus generation and amplification in Sf9 cells

Generation and amplification of recombinant BV vectors was conducted as described by Sung et al. [13, 19]. We have used the following vectors: Bac-CE expressing enhanced green fluorescent protein (eGFP) for cell transduction efficacy assays; Bac-FCVW expressing human VEGF165 flanked by FRT and Bac-FLPo expressing FLP recombinase for VEGF165 expression in mADSC [28]. Stock BV at passage 1 was used for low-multiplicity of infection (MOI) amplification by infecting insect *Sf9* cells in Grace’s insect medium shaker culture. Endpoint assay in *Sf9* culture was used to determine amplified BV titer [19].

### Blood sampling

Blood was sampled from mice at days 0 and 7 of the experiment with a 29G insulin syringe (BD Biosciences (Franklin Lakes, NJ, USA)) by retrobulbar puncture. A sample of 150 μl was drawn during ischemia induction (day 0) and a sample of 300 μl was drawn prior to euthanasia at day 7. Blood samples were stabilized by EDTA and centrifuged (5,000 g, 10 min) in a tabletop 5810R unit (Eppendorf (Hamburg, Germany)) to obtain plasma for ELISA.

### Generation of cell sheets from mADSC

Cell sheets (CS) were generated in uncoated 12-well culture dishes from Corning (Corning,NY, USA). After reaching 80-90 % confluence at P3, mADSC were detached from 100 mm dishes by rinsing with 5 ml of Versene solution and short-term (1–2 min) incubation in 1.5 ml of 0.05 % trypsin/EDTA at 37 °С. By gentle tapping, cells were detached, pipetted and diluted by 6.5 ml of complete DMEM. A 30 μl aliquot was used for trypan blue viability stain and manual cell counts using a hemocytometer. Culture viability > 95 % was typically observed at that stage. Suspended mADSC were centrifuged at 200 × g for 10 min, supernatant was aspirated, and cells were suspended in complete DMEM to obtain 1.0 × 10^6^ cells/ml density prior to seeding.

For CS formation, mADSC were seeded on a 12-well plate at 0.5-1.0-1.5 × 10^6^/well (well surface area = 3.8 cm^2^). After seeding, medium volume was adjusted to 2.5 ml, the plate was agitated by tapping to ensure even cell distribution, and incubated at 37 °С, 5 % СО_2_ for 24 hrs prior to modification by BV.

### BV-transduction of mADSC

For evaluation of BV transduction efficacy we used a monolayer of P3 mADSCs in a six-well plate, which were infected by eGFP-bearing BV (Bac-CE) as follows. Medium was aspirated, cells were washed by PBS twice, and serum-free transduction solution (DMEM, α-MEM, HBSS or PBS) containing Bac-CE (MOI 150 or 75) was added in a total volume of 2.0 ml/well. After that the plate was covered by foil to protect photosensitive BV and incubated at 27 °С on a shaker for 3 or 6 hrs. After transduction cells were washed with PBS, complete DMEM was added, and the cells were incubated for 15 hrs with sodium butyrate (NaBu) to obtain a working concentration of 5 mM. After that, the cells were washed, medium changed to complete DMEM, and the cells were incubated for 48 hrs before fluorescence-activated cell sorting (FACS) to evaluate eGFP expression.

To assess the influence of carbonate buffer on transduction efficacy, mADSC were transduced by Bac-CE (MOI 150) in HBSS mixed with 7.5 % NaHCO_3_ solution to yield a final concentration of 1.0 or 3.0 g/l NaHCO_3_.

For transplantation to mice with induced limb ischemia CS were formed from P3 mADSC in a 12-well plate at 1.0 × 10^6^ cells/well density for 24 hrs. After that they were transduced by Bac-FCVW/Bac-FLPo (MOI 150/15) in HBSS for 6 hrs and treated by 5 mM NaBu (15 hrs) in complete DMEM prior to detachment. Mock-transduced CS and mADSC for injection in dispersed form were treated by a mixture of HBSS and Grace’s medium from uninfected *Sf9* to imitate transduction conditions.

### FACS analysis of eGFP expression in mADSC after BV transduction

BV-transduced mADSC expressing eGFP were incubated for 48 hours, detached, fixed with 1 % formaldehyde in PBS and used for FACS to yield the percentage of eGFP-positive cells and their mean fluorescence intensity (MFI).

### Cell count and survival analysis

To evaluate changes in cell quantity during transduction 25 × 10^3^ of mADSC (P3) were seeded on a 48-well plate in complete DMEM, attached to plastic for 8 hrs, deprived for 4 hrs in serum-free DMEM, and then subject to Bac-CE transduction (MOI 150, 6 hrs) in PBS or HBSS. After transduction cells were washed, part of the wells was used for manual cell counts using a hematocytometer and trypan blue exclusion stain. Complete DMEM with or without NaBu (5 mM) was added to the remaining wells for 15 hours. Afterwards, cells were detached to obtain endpoint cell counts. Untreated mADSC in complete DMEM were deprived for 4 hrs in parallel with experimental wells and served as a control evaluated at the same time points.

### ELISA for human VEGF165

We used the manufacturer’s protocol (R&D Systems (Minneapolis, MN, USA)) for detection of human VEGF165 in CS culture medium and mouse plasma samples by ELISA using the corresponding Quantikine® kit (Cat#DVE00).

### Cell sheet labeling by CMFDA and CM-Dil

CellTracker green CMFDA was added to CS at working 1:2000 dilution to culture medium for 1 hr prior to detachment and transplantation. Suspended mADSC were labeled by CellTracker CM-Dil (Invitrogen) according to the manufacturer’s protocol in PBS. Afterwards, CS or suspended mADSC were washed and observed under a fluorescent microscope prior to transplantation to ensure dye incorporation.

### Detachment of cell sheets by trypsinization

CSs were washed with 2 ml of PBS twice and then once with 2 ml Versene solution. After that, CS were treated with 0.5 ml 0.025 % trypsin/EDTA per well for 15 sec, washed with 2 ml of PBS twice and detached by tapping and manipulation with a plastic disposable tip to induce CS flotation (see Additional file 1: video 1).

### Mouse hind limb ischemia model and CS transplantation

C57/B6 male mice (eight- to ten-weeks old) were narcotized by intraperitoneal injection of 2.5 % avertin solution. All surgical manipulations were carried out in aseptic conditions under a binocular microscope. Unilateral induction of hind limb ischemia was carried out as previously described [29]. Briefly, skin was incised along the midline of the left hind limb and the femoral artery with its branches was ligated between its proximal part and popliteal bifurcation. The blood vessel was excised between upper and lower ligatures with the sciatic nerve kept intact.

After that, the mock-transduced or VEGF165-epxressing CS was transplanted in a drop of PBS to cover the site of excised blood vessels and dried by a cotton ball. Animals that received CS transplantation formed ADSC CS or VEGF-ADSC CS groups (n = 7/group). In the untreated control group (n = 9) the wound was rinsed with PBS and dried. After CS adhesion (1–2 min) and control of hemostasis, the skin was closed with 5–0 silk sutures and the animals were placed in a chamber on a heated pad until full recovery. Additionally, a group of mice (n = 7) was injected with dispersed mock-transduced mADSC diluted in 150 μl HBSS with a total of 1.0 × 10^6^ cells. Cells were delivered in three equal injections of 50 μl each to the anterior tibia muscle, the femoral biceps muscle and the femoral quadriceps muscle. After surgery all animals received a 1.5 ml bolus of warm sterile saline subcutaneously to compensate blood loss.

### Laser Doppler perfusion measurement

Blood perfusion was assessed using a Moor LDI 2.0 system in isoflurane-anesthetized animals after surgery and at days 7 and 14 as previously described. Animals were anesthetized by isoflurane inhalation and then placed on a heating pad for ten minutes under 1-2 % isoflurane/oxygen inhalation for stabilization of anesthesia depth and blood pressure. Perfusion measurements on the plantar surface of the animal’s feet (n = 3-4) were made and data variability was analyzed using Moor image Review software. Readings were taken until three subsequent runs with minimal (<10 %) deviation were obtained. To account for variability among measurements, all ambient light and temperature fluctuation readings were normalized against non-ischemic limbs and expressed as relative perfusion (%).

### Microscopy procedures

Whole-mount microphotographs of the tibia anterior muscle sections were taken with an Olympus binocular microscope at low magnification using a CCD camera and AxioVision 3.1 software from Carl Zeiss (Jena, Germany). High power fluorescent and visible light microimaging of sections was made on a Zeiss Axiovert 200 M fluorescent microscope with Axiovision 3.1 software and a CCD camera.

### Muscle harvest and hematoxylin/eosin staining

At days 7 and 14 animals from test groups were sacrificed by lethal isoflurane inhalation. After skin dissection the femoral quadriceps muscle covered by CS or injected with mADSC was harvested and frozen in TissueTek medium. The ischemic tibia anterior muscle was harvested for necrosis assessment and frozen in TissueTek. Parallel frozen sections (7 μm) were prepared on glass slides and stored at −70 °C. We used routine hematoxylin/eosin staining for necrosis analysis in the tibia anterior muscle and for detection of ADSC in the femoral quadriceps muscle. Stained muscles were photographed as described above and necrotic/infiltrated/viable tissue areas were calculated using the color threshold function in NIH ImageJ freeware. Obtained data were used for subsequent statistical analysis after being normalized to section area.

### Immunofluorescent staining and vessel density analysis

Sections were fixed in ice-cold acetone for 20 min, air-dried and washed in PBS (5 min). All antibodies were diluted in blocking solution consisting of 1 % BSA in PBS. After washing, slides were blocked by 10 % normal donkey serum (30 min), washed, and incubated with rat anti mouse CD31 antibody (1:100, 1 hr). Then slides were washed in PBS (3 × 5 min) and incubated in a mixture of anti rat AlexaFluor®594-conjugated antibodies (1:800) and anti α-SMA FITC-conjugated antibodies (1:50, 1 hr). At the end of incubation slides were washed, counterstained with DAPI (1:20000, 5 min), and mounted.

Microphotographs of sections were taken under 200x magnification in five random fields of view (FOV) per section. Vessel counts were performed blind by two independent persons using NIH ImageJ freeware. Capillary density analysis included CD31-positive structures per FOV; arteriolar counts per FOV were estimated as number of α-SMA-positive vessels with a clearly visible CD31-positive inner layer. Capillary and arteriole counts per FOV were pooled to obtain mean values and for subsequent statistical assessment.

### Ki-67 and cleaved caspase 3 immunofluorescent staining

Sections of the femoral quadriceps muscle covered by CS were fixed in 4 % formaldehyde (20 min), washed in PBS (3 × 5 min), blocked by 10 % normal donkey serum for 1 h, and incubated overnight with rabbit polyclonal antibodies against mouse Ki-67 (1:50) or cleaved caspase-3 (1:50) at 4 °C. After washing in PBS (3 × 5 min), secondary donkey anti rat AlexaFluor®594-conjugated antibodies (1:800) were added for 1 h, slides were rinsed with PBS and nuclei were counterstained by DAPI. Microphotographs were taken with 400× or 630× magnification using immersion oil. Cells positive for Ki-67 and cleaved caspase 3 were counted manually and normalized to nuclei counts per FOV to obtain the percentage of proliferating and apoptotic cells.

### Statistical analysis

Data are expressed as mean ± SD or SEM where appropriate obtained from three or four serial runs per experiment. The statistically significant difference between two groups was determined using a Student’s t-test or Mann–Whitney rank sum U test depending on sample distribution profile. Multiple groups were compared using analysis of variance (ANOVA) with Bonferroni correction for level of significance where required (p < 0.05 was considered significant). Wizard 1.6.2 and Statsoft Statistia 8.0 were used for analysis of obtained data.

## Results

### Rapid formation of cell sheets from mADSC

**Fig. 1:**
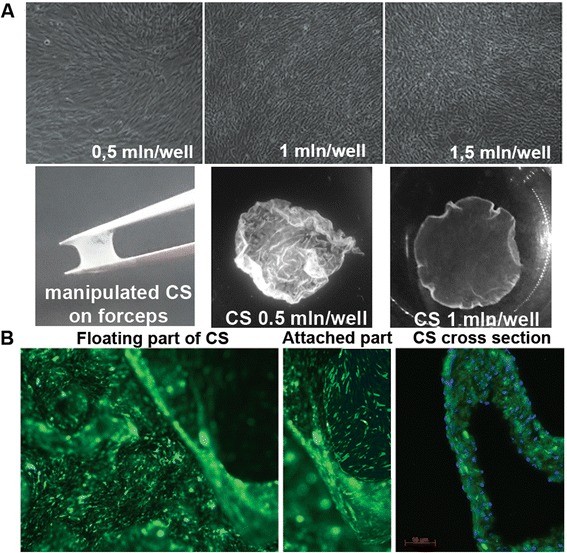
Cell sheet formation and integrity of structure. **a** *Upper row*: images of mADSC seeded at various densities, 100× magnification (phase-contrast); *lower row*: gross view of detached CS on forceps (*left*) and in PBS (*middle* and *right*). **b**. Images of detached CS from 1.0 × 10^6^ of mADSC labeled by CMFDA showing integrity of its floating part (*left*), attached part (*middle*), and its multilayered structure on frozen cross section (*right*, 200× magnification, nuclei stained by DAPI). *mADSC* mouse adipose-derived stromal cells, *CS* cell sheet, *CMFDA* 5-chloromethylfluorescein diacetate, *DAPI* 4′,6-diamidino-2-phenylindole

We observed rapid (within 12–24 hrs) formation of CS from mADSC seeded to a 12-well plate at a density of ≥ 1.0 × 10^6^ cells/well (≈250 × 10^3^ cells/cm^2^). Lower cell density resulted in shattering of CS during trypsinization or transfer to animal muscle. Morphology of cultured mADSC changed drastically at density ≥500 × 10^3^ cells/well and was characterized by condensed, closely attached cells. Interestingly, mADSC formed detachable CS at 1.0 × 10^6^ cells/well within 6 hrs post seeding, but at 500 × 10^3^ cells/well CS required at least 72 hrs of incubation to avoid disintegration and to be manipulated by surgical tools (Fig. 1a).

We stained CS with cytoplasmic CMFDA dye prior to trypsinization to perform visual assessment of floating and attached parts of CS. We found no evidence for CS disruption and established short-term (12–15 sec) treatment with a low (0.025 %) concentration of trypsin/EDTA as an appropriate method for CS detachment (Fig. 1b). To analyze CS structure we prepared frozen cross-sections of CMFDA-labeled CS formed from 1.0 × 10^6^ cells for 24 hrs. Our data suggest the CS has an average thickness of approximately 60–70 μm and consists of three to six layers of cells (counted by DAPI-positive nuclei) (Fig. 1b, right panel).

### Influence of transduction medium and its buffer system on BV-infection efficacy

Since specific surface markers of BV-entry permission are yet to be identified [30, 31], while establishing a transduction procedure one has to optimize conditions depending on cell source, type, and medium used. Initially we analyzed the influence of medium, MOI, and transduction duration using Bac-CE and FACS-analysis of eGFP expression.

**Fig. 2:**
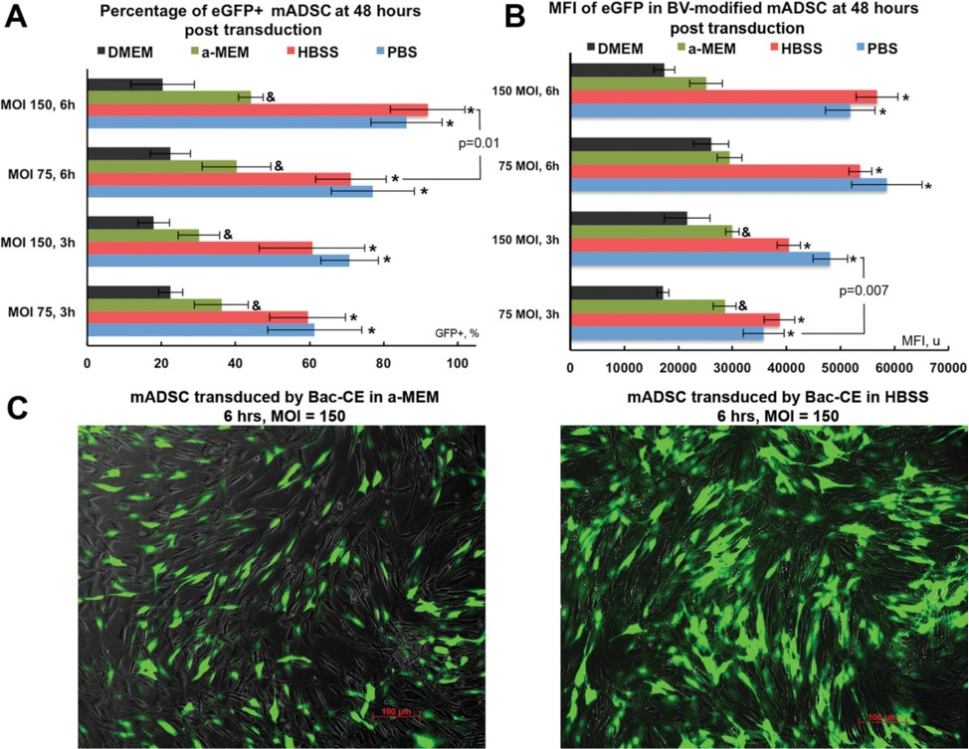
Influence of medium, incubation time and MOI on transduction efficacy and eGFP production. **a** At both durations and MOI, maximum of eGFP-positive cells was found after transduction in HBSS and PBS. **b** eGFP production evaluated by MFI correlated with transduction efficacy and the amount of eGFP-positive cells showing PBS and HBSS to be similarly superior to DMEM and α-MEM. **c** Representative images of mADSC expressing eGFP 48 hours after being transduced by Bac-CE in α-MEM and HBSS. * p < 0.025 vs. DMEM and α-MEM under similar transduction conditions; & p < 0.05 vs. DMEM under similar transduction conditions; Mann–Whitney U test with Bonferroni correction. *MOI* multiplicity of infection, *GFP* green fluorescent protein, *HBSS* Hanks balanced salt solution, *PBS* phosphate-buffered saline, *MFI* mean fluorescence intensity, *DMEM* Dulbecco’s modified Eagle’s medium, *α-MEM* α-minimal essential medium, *mADSC* mouse adipose-derived stromal cells

FACS analysis of mADSC transduced by Bac-CE for 3 or 6 hrs showed that use of PBS and HBSS for virus dilution resulted in a significantly higher percentage of eGFP-positive cells (up to 85-95 %) compared to α-MEM or DMEM (Fig. 2a). We found that MOI influenced the percentage of eGFP-positive cells and in HBSS samples it was significantly higher at MOI 150 compared to MOI 75 (91.9 ± 10.1 vs. 71.2 ± 9.5 %, respectively; p = 0.01).

Similar results were obtained during analysis of MFI reflecting eGFP production. HBSS and PBS were also superior to DMEM and α-MEM (Fig. 2a and b) suggesting higher protein yield. MFI was at a plateau level and did not change with MOI increment in cells transduced by Bac-CE diluted in PBS and HBSS for 6 hrs (See Fig. 2b). After a 3 hr incubation with Bac-CE in PBS we found MFI to be significantly higher at MOI 150, than at MOI 75 (Fig. 2b).

Among tested media, α-MEM and DMEM, which had a lower efficacy, are carbonate-buffered solutions with 2.2 and 3.7 g/l of NaHCO_3_, while PBS and HBSS are based on the buffering capacity of phosphates containing 0.0 and 0.35 g/l of NaHCO_3_. Supplementation of medium with NaHCO_3_ is known to hinder BV entry [32], so we studied its effect on mADSC transduction. Addition of NaHCO_3_ to HBSS (which showed optimal performance) resulted in a drastic drop of eGFP-positive cells_._ Addition of 1.0 g/L of NaHCO_3_ led to the decline of transduction efficacy from 89.5 ± 4.2 % to 44.2 ± 6.5 % (p = 0.009) and at 3.0 g/l NaHCO_3_ efficacy was even lower (29.5 ± 3.7 %). Based on these data, in further cell survival analyses we focused on phosphate-buffered transduction media and used HBSS or PBS for virus stock dilution.

### Cell survival after BV-mediated delivery of eGFP

High protein yield in BV-based procedures can rely on the effects of sodium butyrate (NaBu), which is a non-specific histone deacetylase inhibitor. It induces remodeling of chromatin and can significantly boost protein production yet exerts toxicity at high concentrations [33].

**Fig. 3:**
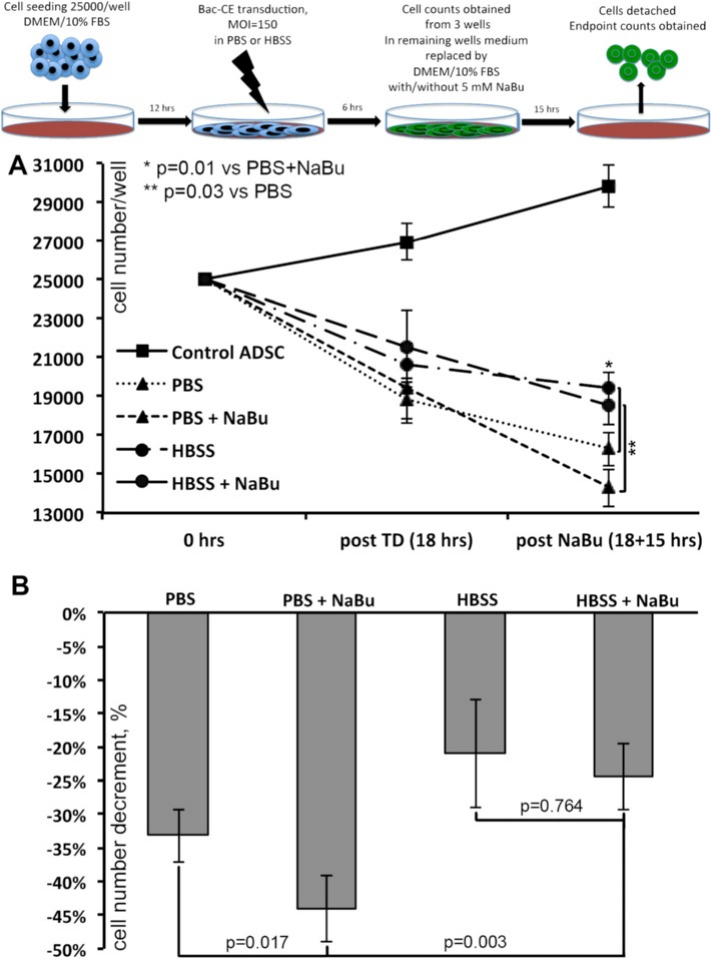
Cell survival after BV transduction in PBS or HBSS and stimulation with sodium butyrate. **a** Cell count dynamics over the time of the experiment shows that use of glucose-containing (1.0 g/L) HBSS results in higher cell survival compared to PBS at endpoint (post NaBu). **b** Cell count decrement at endpoint (18 + 15 hrs) expressed as percentage from initially seeded 25 × 10^3^ cells. Mann–Whitney U test with Bonferroni correction, *BV* baculovirus, *PBS* phosphate-buffered saline, *HBSS* Hanks balanced salt solution

To assess survival of mADSC and effects of NaBu we obtained manual counts of cells transduced in PBS or HBSS with or without subsequent NaBu treatment. Prior to BV-treatment all cells were deprived to limit the effect of ongoing cell proliferation on count results. At the end of transduction by Bac-CE (6 hrs) we observed a similar 15-20 % count decline (Fig. 3a: post TD), which tended to be more prominent after transduction in PBS independently of NaBu treatment. After mADSC were washed and incubated with or without 5 mM sodium butyrate from 15 hrs, cell number reduction was observed in all samples (Fig. 3a: post NaBu). Still, mADSC transduced in HBSS had significantly higher cell numbers than cells treated with Bac-CE diluted in PBS in both NaBu-treated or control medium samples.

Extent of cell count decline was recalculated with the initial 25 × 10^3^ cells as 100 % (Fig. 3b). We found that transduction in more nourished HBSS was able to slightly mitigate effects of NaBu and cell count decrease was similar in NaBu-treated and untreated samples (−24.4 ± 5.3 % vs. -21.1 ± 7.8 %; p = 0.764). Unfortunately, treatment of PBS-transduced cells with NaBu led to a significant drop of cell number compared to NaBu-free samples (−44.1 ± 5.4 % vs. -33.7 ± 4.4 %; p = 0.017), which was not observed in HBSS. Based on these findings HBSS was preferred for transduction of CS prepared for animal procedures.

### Production of human VEGF165 by mADSC CS after BV-mediated gene delivery

**Fig. 4:**
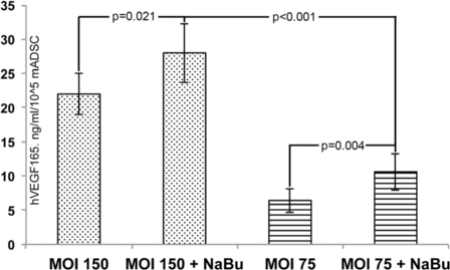
Production of human VEGF165 by mADSC CS after transduction by Bac-FCVW/Bac-FLPo. Culture medium samples were assayed 48 hrs post transduction and VEGF165 concentration was normalized to cell quantity after CS disruption by trypsinization. Mann–Whitney U test. *VEGF165* vascular endothelial growth factor, 165 amino acid isoform, *mADSC* mouse adipose-derived stromal cells, *CS* cell sheet

We have established a protocol for BV gene delivery of VEGF165 to mADSC using HBSS for viral stock dilution with 6 hrs of incubation. Treatment of 1.0 × 10^6^ cell CS from mADSC with Bac-FCVW/Bac-FLP at both tested MOIs (75 or 150) resulted in onset of production of human VEGF165 as soon as 48 hrs post transduction (Fig. 4) with MOI 150 yielding significantly more protein of interest. Still, maximum amounts of VEGF165 at both MOIs was detected after treatment of transduced cells with 5 mM sodium butyrate reaching up to 27.2 ± 4,0 ng/ml/10^5^ cells at MOI 150 (Fig. 4).

### Subcutaneous transplantation of CS induces restoration of hind limb perfusion

**Fig. 5:**
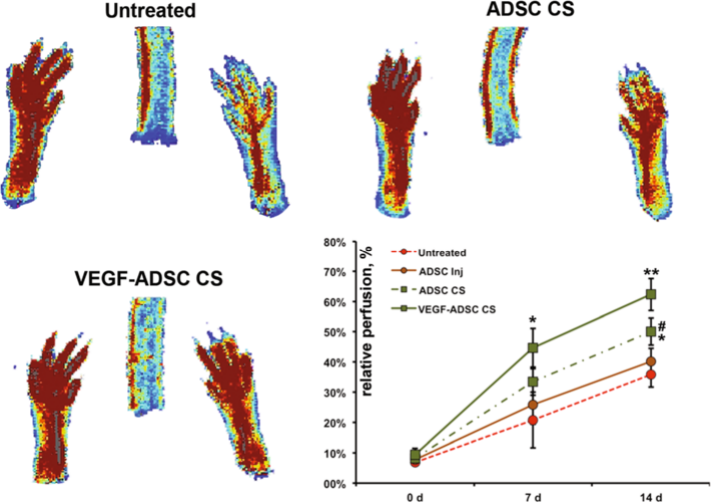
Effect of VEGF-expressing CS, mock-transduced CS or ADSC suspension on perfusion in the limb ischemia model. Presented Doppler images in experiment group animals taken at day 14; chart reflects dynamics of limb perfusion in untreated negative control (n = 9) or animals that received transplantation of VEGF-expressing (VEGF-ADSC CS, n = 7), mock-transduced CS (ADSC CS, n = 7) or suspended mock-transduced mADSC by intramuscular injection (ADSC inj, n = 7). *p < 0.025 vs. untreated control; **p = 0.007 vs. untreated control and p = 0.038 vs. ADSC CS; #p = 0.01 vs. ADSC injection. Mann–Whitney U test. *VEGF* vascular endothelial growth factor, *CS* cell sheet, *ADSC* adipose-derived stromal cells

After mADSC were transplanted to ischemic muscle by CS adhesion or intramuscular injection we observed no limb loss or extensive necrosis throughout the experiment. Transplantation of CS from mADSC (whether it was transduced to express VEGF or not) resulted in increased limb perfusion starting from day 7 (chart in Fig. 5). No significant difference was found at day 7 between VEGF-expressing and mock-transduced CS groups (44.3 ± 11.6 % vs. 35.2 ± 11.1 %, respectively; p = 0.286). At day 7, only the VEGF-ADSC CS group showed significant improvement compared to untreated animals, which had relatively low perfusion of 22.7 ± 8.5 %. By day 7, the ADSC-injection group showed minor positive changes with an average of 26.7 ± 5.3 % (p = 0.36 vs. untreated).

At the end of the experiment (day 14) both CS groups displayed robust improvement of limb perfusion with 62.3 ± 6.8 % in VEGF-ADSC CS and 50.1 ± 6.5 % in ADSC CS groups (p < 0.05 vs. 35.8 ± 6.6 % in untreated). Importantly, we found that endpoint perfusion in the VEGF-ADSC CS group was significantly higher than in mock-transduced CS (p = 0.038), which can be accounted for VEGF165 production after BV modification. In our experiment we found that by day 14 injection of dispersed ADSC resulted in a moderate blood flowincrement (40.2 ± 4.8 %) without statistical significance compared to untreated mice (p = 0.19). Moreover,animals that received injection of dispersed ADSC showed lower perfusion than animals that underwentADSC CS transplantation (p = 0.01).

### mADSC are retained in skeletal muscle at day 14 after transplantation by injection or CS delivery

**Fig. 6:**
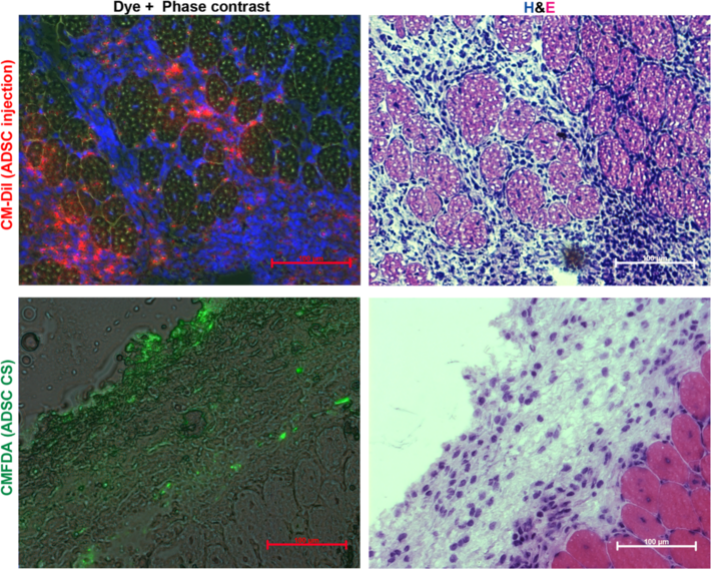
Retention of mADSC after subcutaneous transplantation of CS or intramuscular injection of dispersed cells. Prior to transplantation CS were labeled by CMFDA (green) and dispersed mADSC – by CM-Dil (red) vital dyes. At day 14 after transplantation animals were sacrificed and skeletal muscle samples were used for detection of ADSC retention at the site of delivery (on the surface of the femoral quadriceps muscle for CS or within the anterior tibia muscle for injected mADSC). In the CMFDA-labeled CS, DAPI stain was omitted due to significant signal quenching after washing of fresh sections in saline or fixation. Corresponding sections were stained with H & E to evaluate tissue status. *mADSC* mouse adipose-derived stromal cells, *CS* cell sheet, *CMFDA* 5-chloromethylfluorescein diacetate, *DAPI* 4′,6-diamidino-2-phenylindole, *H & E*, hematoxylin and eosin

During necropsy skin at the site of surgery was dissected and carefully removed. We found that CS were adherent to muscle surface, but not to dermal layers, which allowed us to separate a part of the ,muscle together with CS. At gross examination transplanted CS were described as flat, opaque oval or disc-shaped structures with uneven or barely visible margins covering the area of the excised anterior femur. Microscopy of muscle sections at day 14 revealed CMFDA-positive cellular layers adherent to the muscle (Fig. 6, lower row). As for injected mADSC labeled by CM-Dil we found them to reside within skeletal muscle resembling cellular infiltrates located around myofibers in the middle portion of the section near the injection site (Fig. 6, upper row). Corresponding sections were also stained with H & E to show that CS have a multilayered structure with a thickness off 50–100 μm resembling a cross-section of detached CS (Fig. 1b). Sections stained with H & E allowed us to detect adherent grafts and were used for orientation within the tissue sample (Additional file 2: Figure S1).

### Induction of capillary angiogenesis in skeletal muscle after CS transplantation

**Fig. 7:**
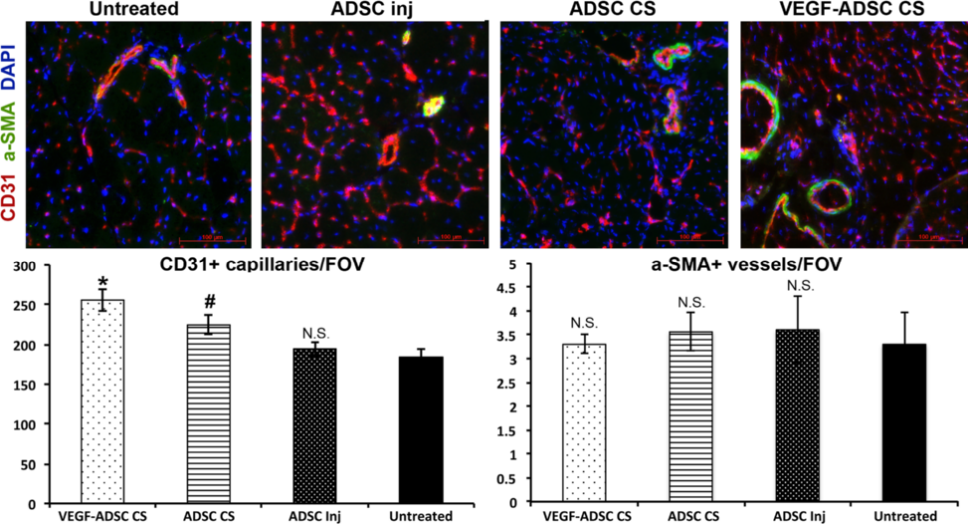
Analysis of blood vessel density in skeletal muscle sections from study group animals. Histological studies of the anterior tibia muscle were performed in samples harvested at day 14 of the experiment (n = 6/group): *upper row* – representative images of muscle sections from study group animals stained for CD31 and α-SMA (magnification × 200); *lower row* – graphical presentation of blood vessel density analysis with average group values per FOV; *p < 0.05 vs. untreated; **p < 0.025 vs. ADSC and Untreated; N.S. – not significant; Mann–Whitney U test with Bonferroni correction. α-*SMA*, α-smooth muscle actin, *FOV* field of view, *ADSC* adipose-derived stromal cells

Histologic assessment of the anterior tibia muscle extracted at day 14 showed that capillary density was significantly increased in both CS-treated groups compared to untreated controls (Fig. 7). Analysis of sections showed that in the ADSC CS group there was an average of 224.3 ± 11.8 capillaries vs. 184.3 ± 10.6 in untreated mice (p < 0.001). Maximum capillary density was observed in the VEGF-ADSC CS group with an average of 255.6 ± 13.7 (p = 0.006 vs. ADSC CS and p < 0.001 vs. the untreated control). Mice that received an injection of dispersed ADSC had significantly lower capillary density compared to ADSC CS animals with an average of 194.5 ± 8.8 (p = 0.01 vs. ADSC CS). Moreover, to our surprise, we found that after injection of dispersed ADSC capillary density was just slightly higher, than in untreated mice (p = 0.32 vs. corresponding control).

Another important characteristic of angiogenesis is collateral remodeling or *de novo* formation of larger blood vessels with mural α-SMA-positive smooth muscle cells and inner lining of CD31-positive endothelium (Fig. 7). Throughout the experimental groups we observed similar density of α-SMA-positive vessels per FOV: 3.31 ± 0.6 in untreated, 3.56 ± 0.4 in ADSC CS, 3.5 ± 0.61 in ADSC injection group, and 3.41 ± 0.21 in VEGF-ADSC CS (p > 0.05 in Kruskall-Wallis test). Thus, we found no increment of arteriogenesis, which was possibly attributed to a relatively early study endpoint (14 days).

### Reduction of muscle necrosis and inflammation after ADSC delivery

The ability to prevent muscle necrosis is a crucial feature of therapeutic intervention and evidence for adequate restoration of blood supply.

**Fig. 8:**
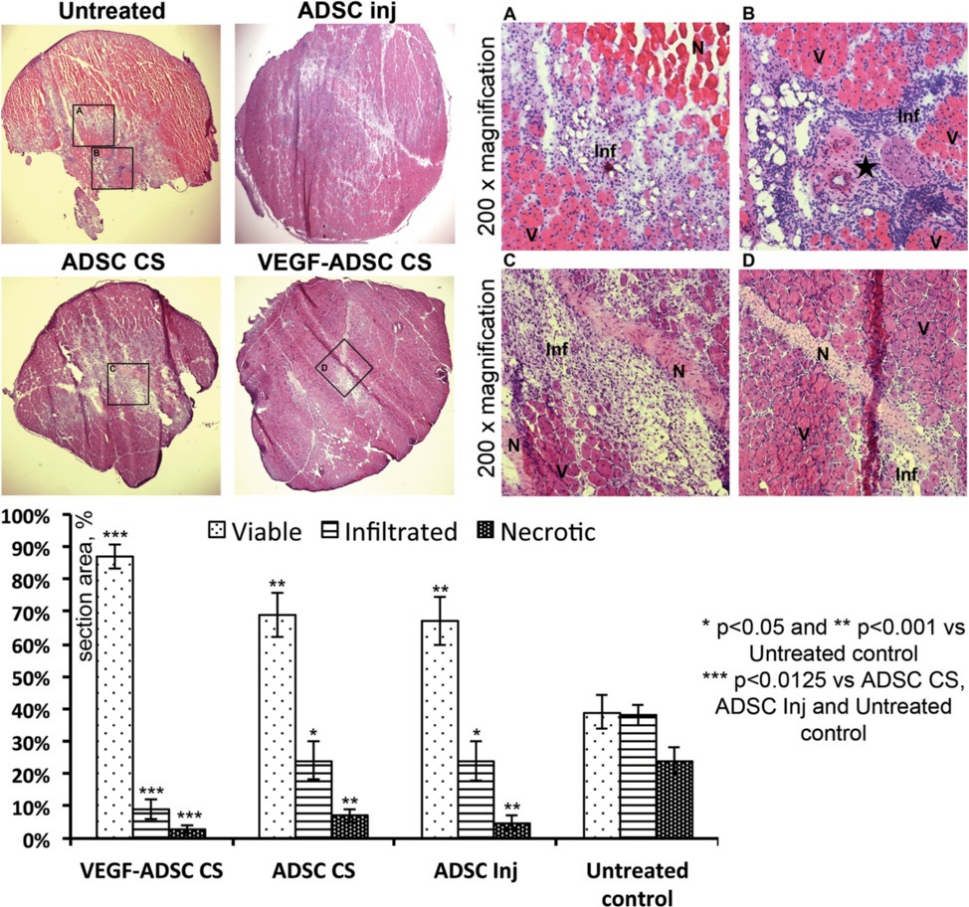
Reduction of skeletal muscle necrosis and infiltration after delivery of ADSC. *Upper left* – Whole-section images of hematoxylin/eosin-stained muscle from corresponding study group; *Upper righ*t (**a**-**d**) Microphotographs representing histological changes in areas outlined in the *upper row*. **a** Border area with necrotic fibers (N), inflammatory cells infiltrate (Inf) and viable tissue (V). **b** Artery-vein-nerve (*star*) complex with expressed inflammatory extravasation. **c** and **d** Fibrotic tissue on site of resolved ischemic necrosis. *Lower row* Graphic presentation of morphometry results; ANOVA and Student t-test with Bonferroni correction. *ADSC* adipose-derived stromal cells, *ANOVA* analysis of variance

For analysis we used the anterior tibia muscle, which suffers from severe ischemia after anterior femur excision. Morphometric assessment of sections (details of procedure can be found in Additional file 3: Figure S2) indicated that in untreated controls the ratio of areas covered by viable/infiltrated/necrotic tissue was close to 1:1:1 (chart in Fig. 8). In the mADSC groups we observed a drastic reduction of necrosis span from 26.3 ± 4.3 % in untreated down to 6.9 ± 2.0 % and 5.7 ± 1.9 % in CS and injection groups, respectively; p < 0.001 for both). Decrease of necrosis in VEGF-ADSC CS was even more pronounced and this group had the lowest number of necrotic fibers with a more than two-fold decrease compared to the CS and injection groups (2.9 ± 1.3 % vs. 7.2 ± 2.1 %; p = 0.005). Infiltration by inflammatory cells was also reduced in the ADSC CS and injection groups resulting in 24.2 ± 5.7 % and 23.8 ± 5.1 %, respectively, which was in both cases lower than in untreated control s(37.4 ± 4.6 %; p < 0.05 for both).

Moreover, in all ADSC-treated groups we observed a significant increase in viable tissue, which indicated a protective effect. The VEGF-ADSC CS group was characterized by the highest amount of viable and healthy tissue compared to other ADSC CS, injected ADSC or untreated control groups (chart in Fig. 8).

### Histology assessment of implanted CS vascularization

Engraftment relies on transplant vascularization; thus, appearance of blood vessels within CS can serve as a sign of successful implantation. Staining of the femoral quadriceps muscle covered by CS revealed a number of capillary-sized CD31-positive vessels within the mADSC mass and sporadic α-SMA-positive larger vessels. Vascularization of CS was found in all analyzed specimens (n = 2-3/group) with CS located using parallel H & E slides or fresh sections of CMFDA-stained CS.

A number of muscle fibers underlying CS had centrally located nuclei indicating young skeletal muscle fiber formation [34]. This suggests that CS-covered muscle is capable of restoring its morphology after initial damage during surgical manipulation and subsequent ischemia.

### Cell proliferation and apoptosis in transplanted CS from mADSC

Cell fate after transplantation is always of crucial interest, although its tracking is rather methodologically complicated. We stained sections of femoral quadriceps muscle with adherent CS for proliferation (Ki-67 antigen) and apoptosis (cleaved caspase 3).

Figure 10a demonstrates CMFDA-labeled Ki-67-positive cells, which are likely to be proliferating transplanted mADSC as well as intruder Ki-67-positive cells without CMFDA label. Proliferating cells of both types were found within CS in both ADSC CS and VEGF-ADSC CS animals at approximately equal quantities at day 14 after CS transplantation indicating proliferation of transplanted CMFDA-positive mADSC as well as invading host cells. Quantitative analysis of cells positive for Ki-67 in acquired images indicated no significant difference in the prevalence of proliferating cells between ADSC CS and VEGF-ADSC CS groups (6.28 ± 2.1 % vs. 6.01 ± 1.2 %, respectively; p > 0.05)

As for evaluation of apoptosis responsible for decline of transplanted cells, we used cleaved caspase 3 antibody (Fig. 10b). Cells showed both nuclear and cytoplasmic localization of active caspase 3 indicating different stages of apoptosis, yet we did not find any difference of apoptotic cells in ADSC CS and VEGF-ADSC CS (12.5 ± 2.7 % vs. 11.9 ± 4.2 %, respectively; p > 0.05).

### Transplantation of VEGF-expressing CS does not result in systemic dissemination of human VEGF165

In the VEGF-ADSC CS group ELISA of mouse plasma drawn at day 7 did not reveal any detectable quantity of human VEGF165. Values were below the level of detection of the ELISA kit (not shown) and optical densities were identical to values observed at day 0 (pre-transplantation baseline). Thus, no evidence for systemic circulation of human VEGF165 was found in our model.

## Discussion

In the last decade cell therapy of limb ischemia has been extensively developed exploiting different cell types and a variety of delivery methods. Still, the transplantation procedure and its therapeutic efficacy remain issues despite feasible access to skeletal muscle (compared to myocardium or brain) and sophisticated guidance and application techniques we dispose at the moment [35]. Our work reports a rapid and reproducible method for generation of CS from ADSC and suggests aprocedure for baculoviral expression of VEGF to enhance their angiogenic efficacy.

Formation of CS requires optimal conditions partially depending on species due to cell size differences between mammals (e.g., human ADSC are nearly three times as big as mouse) and matrix synthesis rate. Our data confirmed the role of optimal seeding density (Fig. 1a and b): formation of CS from mADSC was observed at 1.0-1.5 × 10^6^ cells/well of a 12-well plate (≈250-375 × 10^3^ cells/cm^2^), which allowed the generation of a transplant with a surface of 0.8-1.0 cm^2^. The latter was smaller than the well’s bottom area due to surface tension-induced retraction of CS. At lower densities formation of CS from mADSC may require up to five days [24] and after seeding of < 500 × 10^3^ cells/well in a 12-well plate we incubated them for at least three days to obtain a detachable structure (data not shown). Thus, both approaches led to generation of CS from mADSC, but a higher seeding density allowed reduction of time required for CS to form and become detachable. However, rapid generation relies on a large number of cells, which is feasible for ADSC, but for other cell types, which are not available in abundance or have lower proliferation rate, this can be an obstacle and turn attention to long-term incubation.

Cell sheets from mADSC were detached using short-term trypsinization without loss of integrity and comprised a solid structure of approximately 50–70 μm thickness, which accounts for three to six layers of cells (Fig. 1). Most studies in the field exploit commercial or in-house made thermoresponsive culture dishes [36, 37] where detachment of CS occurs due to polymer coating that changes its hydrophilic properties depending on temperature. We had to omit use of thermoresponsive dishes because the protocol for BV transduction included 6 hrs of incubation at 27 °C, which resulted in loss of adherence. This methodical hurdle was circumvented by application of short-term (10–15 sec) trypsinization and also reduced the procedure’s cost by using routine laboratory equipment to detach and manipulate the CS (Fig. 1 and Additional file 1: Video 1).

We established a protocol for effective BV-transduction of mADSC in HBSS with subsequent treatment by sodium butyrate. Transduction of mammalian cells by BV is influenced by a number of factors including temperature, presence of serum or other supplements and transduction medium buffer system.

FACS-analysis of eGFP expression showed that BV-transduction in PBS or HBSS resulted in up to 90 % efficacy after 6 hrs of incubation at MOI = 150 (Fig. 2a). Our data support the use of low-NaHCO_3_ media for viral transduction of mADSC in the same manner as it was observed by Shen et al. [32]. In our study use of α-MEM and DMEM for viral stock dilution led to reduced transduction efficacy, which correlated with higher NaHCO_3_ content in these media known to hinder BV transduction of mammalian cells. The negative influence of NaHCO_3_ is attributed to its ability to reduce the amount of BV entering the cell without significant influence on viral particles stability or binding to the cell membrane [32].

Cell survival after BV transduction was assessed using eGFP-expressing vector to avoid mitogenic or pro-survival effects of VEGF165 and showed that use of HBSS is preferable to PBS. Despite containing only 1.0 g/L D-glucose HBSS significantly attenuated toxicity of NaBu added to the cells to boost protein production (Fig. 3). Thus, we suggest that even minimally nourished HBSS mitigated stress that mADSC underwent during transduction and NaBu treatment so we used it in further procedures.

Expression of human VEGF165 in rodent ADSC using the Bac-FCVW/Bac-FLPo system has showed its efficacy in a rabbit model of myocardial infarction with a prolonged (>1 month) expression period *in vitro* [24] and minimal immunogenicity *in vivo* [38]. In the current work this approach has been successfully applied for modification of high-density CS from 1.0 × 10^6^ mADSC. Our experiments suggest that maximum human VEGF165 output was achieved after transduction by Bac-FCVW/Bac-FLPo MOI of 150/15 with subsequent NaBu treatment resulting in VEGF165 content up to 25–27 ng/ml/10^5^ cells (Fig. 4). Still, protocols resembling ours are not limited to application of BV for ADSC – due to the mechanism of BV entry after certain tests it can be adapted for modification of other cell types. BV-mediated transduction of chondrocytes [39], endothelial, cancer and epithelial cells [40] has been reported to result in significant expression of the delivered gene. During development of new approaches, NaBu treatment can be omitted in case of prominent deteriorating effects on cell survival yet in ADSC we managed to establish a “tempered” protocol when application of appropriate medium (HBSS) and other transduction conditions resulted in acceptable cell survival along with peak VEGF165 production.

The method of transplantation we used requires routine laboratory equipment for detachment and transfer of CS and manipulations within the surgical wound were performed in a drop of saline to reduce mechanical damage. High adherence of CS to skeletal muscle allowed omitting any additional material (e.g., fibrin glue). Furthermore, the stickiness of CS was supported by histology findings at day 14 that showed close contact of dye-labeled CS with skeletal muscle (Fig. 6 and Additional file 2: Figure S1). Interestingly, in CS-covered muscle we observed a number of young myofibers underlying the mADSC mass, which raises a question whether myogenesis has been supported by CS or was induced by surgical and ischemic injury to the muscle.

The conventional method for ADSC delivery is injection of cells suspended in an isotonic vehicle solution. Evaluation of dispersed mADSC engraftment after intramuscular injection revealed that dye-labeled cells resided between myofibers at day 14 (Fig. 6). Labeled mADSC located within the injection site were numerous indicating engraftment and survival, which is in accordance with previous findings in BALB-C nude mice [8].

In the described case, the putative advantage of CS besides transplantation of ADSC with matrix proteins is the allocation of cells to the peripheral region of the muscle (Fig. 5 and Additional file 2: Figure S1) where the impact of inflammation and ischemia is not so prominent. Indeed, in examined muscle specimens the majority of viable tissue was located at the periphery, whereas infiltration and necrosis occurred in the middle portion (Fig. 8). In our experiments with BALB-C mice suffering from extreme ischemia after surgery due to weak collateral vascularization we found all central parts of the section to consist of necrotic tissue in different stages of disruption [8]. Thus, we may speculate about a more “tranquil” environment for ADSC delivered on the muscle surface compared to cells injected inside the ischemic tissue. However, injection of dispersed cells is feasible in certain cases and can be easily adjusted for cell number, injection count, and location and can be repeated over time.

Doppler measurements of limb perfusion showed that transplantation of mock-treated CS led to a significant improvement of blood flow compared to untreated controls at day 14 and we also found ADSC CS to be superior to injection of an equivalent dose of cells in terms of limb perfusion (Fig. 5). Even higher perfusion was observed in BV-transduced mADSC expressing VEGF165 suggesting that modification of CS improved its therapeutic efficacy and resulted in a better functional outcome with relative perfusion reaching ≈ 60 %. To our surprise, we found no significant improvement of limb perfusion in animals injected with ADSC despite histology studies revealing engraftment of injected cells. In this case, a possible reason underlying this discrepancy may be duration of follow-up period. In our previous studies with injection of cell suspension to ischemic limbs we found maximum difference between control and treated mice at 20–21 days of experiment. At this time point, spontaneous reperfusion responsible for restoration of blood supply in untreated animals was at the plateau while treated animals showed improvement [8, 29].

Overall, period of observation confines us to certain boundaries and we cannot make claims about the inefficiency of injection delivery. The conclusion that can be drawn from our study is that delivery of ADSC CS to ischemic limbs induces a faster perfusion rise compared to dispersed ADSC. Still, further observation time points are required to fully characterize the efficacy of both methods.

In limb ischemia models perfusion correlates with blood vessel density and we assessed the influence of CS transplantation on capillary and arteriole counts. We found capillary-sized vessels increased in numbers after transplantation of mock-transduced mADSC CS compared to untreated controls (Fig. 7). Delivery of mADSC by injection showed no significant effect on capillary density and was inferior to CS delivery. This finding was quite surprising as paracrine effects of ADSC are known to induce angiogenesis within tissue, matrix, and other animal models of vessel growth.

Maximum increase in CD31+ capillary counts was found in animals that received transplantation of VEGF165-expressing CS, which may be accounted for production of angiogenic growth factor known to induce sprouting and vascularization [8, 41]. In these specimens capillary counts were significantly higher than in mice that received mock-transduced CS subcutaneously. Observed changes in capillary density are classic evidence of angiogenesis stimulation, accepted since early work in this field [42]. Still, we did not find any evidence for enhancement of arteriogenesis assessed by α-SMA+ blood vessels in any study group (Fig. 7). This finding emphasizes importance of expanded observation time-frame as far as stabilization of largerblood vessels may take up to three to four weeks in rodents [43].

Despite the obvious role of larger blood vessels for perfusion, capillaries are known to be of crucial importance for tissue nutrition [44] and do have an impact on blood flow measured by Doppler-based methods [45]. Another mechanism of blood flow restoration relies on collateral formation/remodeling, which may occur at the proximal part of the limb and cannot be evaluated using laser Doppler.

Observed improvement of blood flow was expected to enhance muscle nutrition, which prompted histological evaluation of the anterior tibia muscle (Fig. 8). ADSC CS group animals showed reduced necrosis and infiltration compared to untreated controls, whereas mice that received VEGF-ADSC CS showed significant improvement compared to untreated CS. Attenuation of necrosis is an important endpoint in experimental [8, 29, 46] and clinical studies on limb ischemia therapies [47, 48], thus, this piece of evidence obviously supported the efficacy of the developed technique. Conventionally used injection of ADSC significantly reduced necrosis as well as muscle infiltration (Fig. 8), which indicates that the antiapoptotic effect of this cell type takes place early after delivery and may prevent necrosis and tissue disruption even when vascularization has not yet been established. The latter statement relies on lack of significant changes in perfusion and vessel density in ADSC injected animals. Still, it seems that when the cells are injected and, thus, delivered to the epicenter of ischemia - the central portion of the muscle – they manage to induce a paracrine-mediated response and reduce the number of necrotic fibers.

**Fig. 9:**
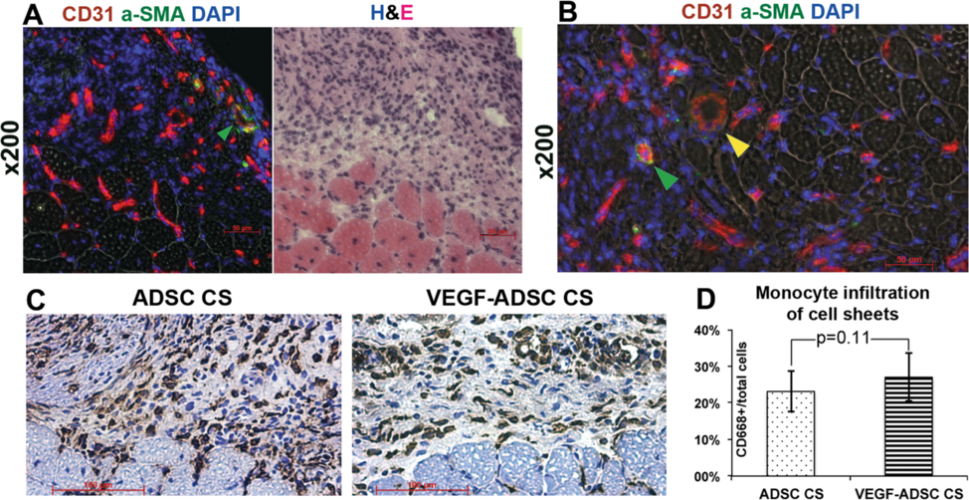
Immunostaining for vascularization and monocyte invasion to transplanted CS. Images were acquired from CS and skeletal muscle sections extracted at day 14 of the experiment and stained for blood vessel and monocyte antigens. **a** Representative image of blood vessels within CS and corresponding H & E staining. **b** Representative image of larger blood vessels within transplanted CS (*green arrow* - α-SMA-positive blood vessels; *yellow arrow* - α-SMA-negative). **c** Microphotographs illustrating CD68+ monocyte infiltration (DAB-visualized) to mock-transduced of VEGF-expressing CS. **d** Graphic presentation of monocyte counts within CS from corresponding study groups; Mann–Whitney U test. *CS* cell sheet, *H & E* hematoxylin and eosin, α-*SMA α*-smooth muscle actin, *DAB* diaminobenzidine, *VEGF* vascular endothelial growth factor

Transplantation of cellular grafts may induce an immune response by the host and we evaluated infiltration of CS by monocytes using immunohistochemical staining for CD68. Monocytes (and their mature forms – macrophages and dendritic cells) are known to play a pivotal role in resorption/phagocytosis, but also drive regeneration and control inflammation [49, 50]. Moreover, despite that the stromal-vascular fraction may contain 10-15 % of the monocytes [51], passaged ADSC are CD68-negative and, thus, evaluation of monocyte infiltration would characterize host response to delivered CS. We found up to 25 % of cells within the CS were CD68-positive (Fig. 9c-d) indicating their monocyte lineage and this figure was similar in both BV-treated and mock-transduced constructs. This was an encouraging finding showing that treatment of cells by BV using our procedure did not enhance the monocyte-mediated immune response to transplanted cells. Moreover, VEGF165 itself may be characterized as a cytokine with certain inflammation-driving modality [52], yet we found no evidence for increased monocyte invasion in VEGF-ADSC CS. Of course, immune response to a graft may involve other cell types, especially CD4+ and CD8+ T-cells, but our previous data in a model of bone defect treated by BV-transduced ADSC showed no significant difference in infiltration by these immune cells [38]. Overall, these findings require additional investigation in a more appropriate model as far as use of inbred strain mice, which are close to the sibling state, does not imitate allogeneic transplantation.

Retention of CS was found at days 7 and 14 and was accompanied by vascularization of transplanted cell mass by capillary (some had visible lumen) and sporadic α-SMA-positive blood vessels in most specimens analyzed (Fig. 9). Formation of functional blood vessels in CS has been previously observed in constructs from endothelial cells [53, 54] and cardiac primitive cells [55]. To our knowledge, this is the first report of blood vessel formation within non pre-vascularized ADSC-based CS in a model of ischemic pathology. Moreover, the short-term CS formation used in our protocol and use of non-angiogenic conditions (DMEM/10 % FBS, normal O_2_ pressure, etc.) marginally excludes the possibility of spontaneous pre-vascularization of CS during *in vitro* preparation. The question to be addressed is the origin of the CD31-positive endothelial cells in the CS. Host blood vessel in-growth is the most obvious answer, while ADSC are known to have a limited capacity for endothelial differentiation [56] or can contain a small fraction of endothelial progenitor cells that may contribute to vessel formation [57, 58].

**Fig. 10:**
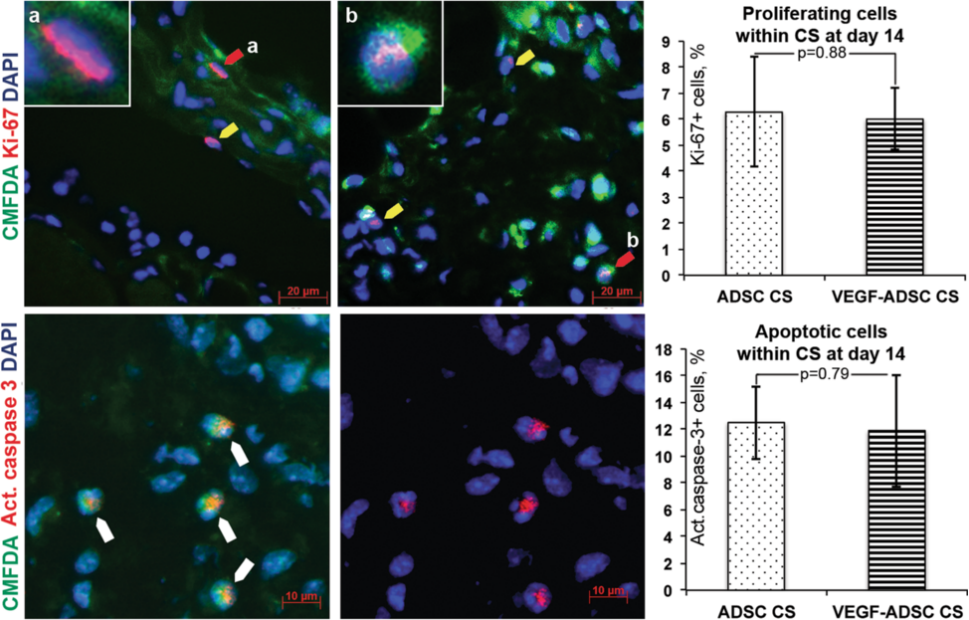
Proliferation and apoptosis of cells within subcutaneously transplanted CS. CS were stained by CMFDA and transplanted to mice subcutaneously. At day 14, CS adherent to the femoral quadriceps muscle was extracted and stained for Ki-67 and cleaved caspase 3. *Upper row left* and *middle* – CMFDA-positive (*red arrows*) and negative (*yellow arrows*) cells with Ki-67+ nuclei indicating proliferation. Magnification 400×. *Lower row left* and *middle* – representative images of cells positive for cleaved caspase 3 (*white arrows*). Magnification 630×. *Right column* graphs illustrate results of manual count of proliferating and apoptotic cells in mock-transduced and VEGF-expressing CS; Mann–Whitney U test. *CS* cell sheet, CMFDA 5-chloromethylfluorescein diacetate, *VEGF* vascular endothelial growth factor

We also obtained evidence for limited (6-7 % of total number) proliferation of cells within CS, which was not confined to transplanted mADSC. We found a number of CMFDA-negative proliferating cells, which are likely to be dividing host cells that invaded CS during engraftment (Fig. 10). As for apoptosis prevalence, it was approximately 10-12 % in both – mock-transduced and VEGF165-expressing CS (Fig. 10). To explain lack of difference between mock-transduced and VEGF-expressing CS we may refer to our published *in vitro* data showing VEGF165 production reduces 12–14 days after transduction, [24] so growth factor may not have had an impact on cell fate.

Observed vascularization of CS stressed in our study provides mechanistic support for the efficacy of CS. We can propose that paracrine activity of ADSC, which is considered to be a driver of its angiogenic and regenerative capacity [40, 59], may have an extended impact area due to growth factor uptake through formed vessels. This may explain stimulation of angiogenesis and reduction of necrotic alterations in the distal part of the limb we used for histology evaluation. Another point supporting a paracrine mode of action in the CS-based protocol is increment of its efficacy after VEGF165 expression in the absence of reduced apoptosis or increased proliferation of transplanted mADSC. Because cell secretome was the only parameter manipulated in this case, it is very likely that growth factors and cytokines could be the main mediators of efficacy.

Subcutaneous implantation of modified or xenogenic CS has been utilized for creating cellular factories producing FVIII in hemophiliac mice [54] or insulin in diabetic SCID mice [60, 61]. In these works the authors observed a significant improvement of blood clotting (with FVIII appearance) and euglycemia (with xenogenic C-peptide detectable) suggesting systemic uptake of secreted factors from CS.

On the one hand, the abovementioned results provide support for the possible concept of uptake of proteins secreted by CS and, thus, may explain the efficacy of distant transplantation of CS. On the other hand, for VEGF165 this could be considered a side effect due to its tumor-activating potential and possible influence on pathologic angiogenesis in retina, skin, etc. [62]. We performed ELISA of plasma from mice at day 7 post-transplantation of VEGF-expressing CS and did not detect systemic circulation of human VEGF165.

The preliminary format of this research has also revealed certain limitations listed below and hinting at further study directions:

1. host/donor origin of vascular cells within CS – the ADSC population mainly contains cells that carry CD31 or may undergo endothelial differentiation [63]; this requires clarification. This point can be addressed using a sex mismatch approach in further studies. Our data also indicate that in angiogenesis models even CD31-negative ADSC may incorporate (unpublished) or locate adjacent to vascular structures acting as pericytes [63];
2. animal study duration – chosen terms of 14 days were optimal for early stage evaluation of necrosis and CS retention, yet extension of the experiment’s time-frame would expand our knowledge of both cell fate and CS effects on perfusion values and vessel density;
3. syngeneic transplantation applied in the study is a widely used model for autologous-based procedures in recent years. However, allogeneic transplantation is easier to scale-up and, thus, properties of allogeneic CS may be of interest for development of clinically relevant procedures. These studies would also provide more valuable data on graft-host interaction;
4. ADSC grafting is donor-dependent so it could of interest to evaluate the influence of aging, chronic diseases and other factors on CS formation and their regenerative properties [64].

## Conclusions

Our study provides a rapid protocol for ADSC cell sheet generation using routine equipment and optimized conditions for ADSC baculoviral transduction to express VEGF165. This modification results in significant improvement of CS therapeutic activity and stimulation of angiogenesis in ischemic skeletal muscle. Subcutaneous transplantation of unmodified CS to ischemic limb was an effective method for angiogenesis stimulation and tissue protection. We suggest a paracrine mode of action for this procedure and report no issues with systemic dissemination of VEGF165. Cell sheets from ADSC reside subcutaneously for at least 14 days and undergo vascularization, infiltration by monocytes indicating interaction between graft and host. We found no evidence for improved proliferation or apoptosis prevalence in VEGF-expressing CS.

## Additional files

Additional file 1: Video 1. Detachment and manipulation of cell sheets from mADSC. Brief introduction to two critical points in ADSC CS preparation – detachment using a pipette tip and manipulation in PBS using a cell-strainer mesh glued to a steel spatula. (MP4 19296 kb) Additional file 2: Figure S1. Examples of hematoxylin-eosin stained sections of mADSC CS attached to mouse muscle. Low magnification images show presence of CS on the femoral quadriceps muscle at day 14 (black arrows). (TIFF 3564 kb) Additional file 3: Figure S2. Illustration for principle of morphometry adopted in tissue section studies. Highlighted regions (bright red) of necrotic and infiltrated tissue in a section of the tibial anterior muscle indicating color threshold selection of regions used for morphometry (hematoxylin/eosin staining). NIH ImageJ freeware for Mac used for processing. (TIFF 4432 kb)

## Abbreviations

ADSC: Adipose-derived stromal cells
α-MEM: α-minimal essential medium
α-SMA: α-smooth muscle actin
BSA: Bovine serum albumin
BV: Baculovirus
CMFDA: 5-chloromethylfluorescein diacetate
CS: Cell sheet(s)
DAPI: 4′,6-diamidino-2-phenylindole
DMEM: Dulbecco’s modified Eagle’s medium
ELISA: Enzyme-linked immunosorbent assay
FACS: Fluorescence-activated cell sorting
FBS: Fetal bovine serum
FOV: Field of view
GFP: Green fluorescent protein
H & E: Hematoxylin and eosin
HBSS: Hanks balanced salt solution
mADSC: Mouse adipose-derived stromal cells
MFI: Mean fluorescence intensity
MOI: Multiplicity of infection
NaBu: Sodium butyrate
PAD: Peripheral artery disease
PBS: Phosphate-buffered saline
RT: Room temperature
SCID: Severe combined immunodeficiency
SD: Standard deviation
VEGF165 or VEGF: Vascular endothelial growth factor, 165 amino acid isoform
