# Supplementary figures and images for: Enhanced angiogenesis in ischemic skeletal muscle after transplantation of cell sheets from baculovirus-transduced adipose-derived stromal cells expressing VEGF165
Source: Stem Cell Res Ther. 2015 Oct 26;6:204. doi: 10.1186/s13287-015-0199-6 (PMC4620646; doi:10.1186/s13287-015-0199-6)

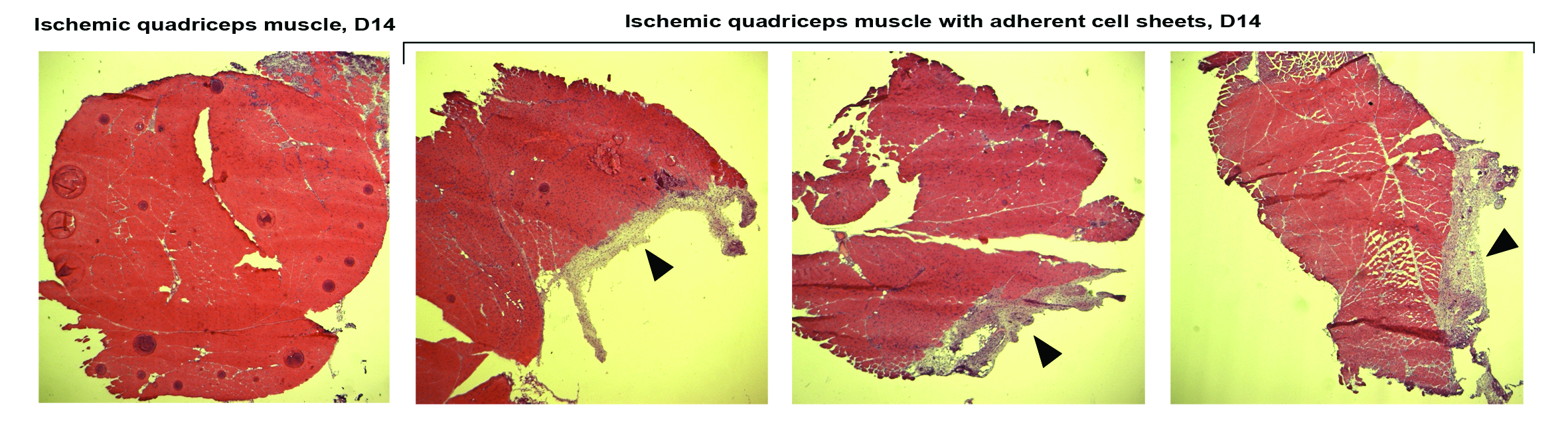

Supplement: Additional file 2: Figure S1. — Examples of hematoxylin-eosin stained sections of mADSC CS attached to mouse muscle. Low magnification images show presence of CS on the femoral quadriceps muscle at day 14 (black arrows). (TIFF 3564 kb) [file 13287_2015_199_MOESM2_ESM.tif]

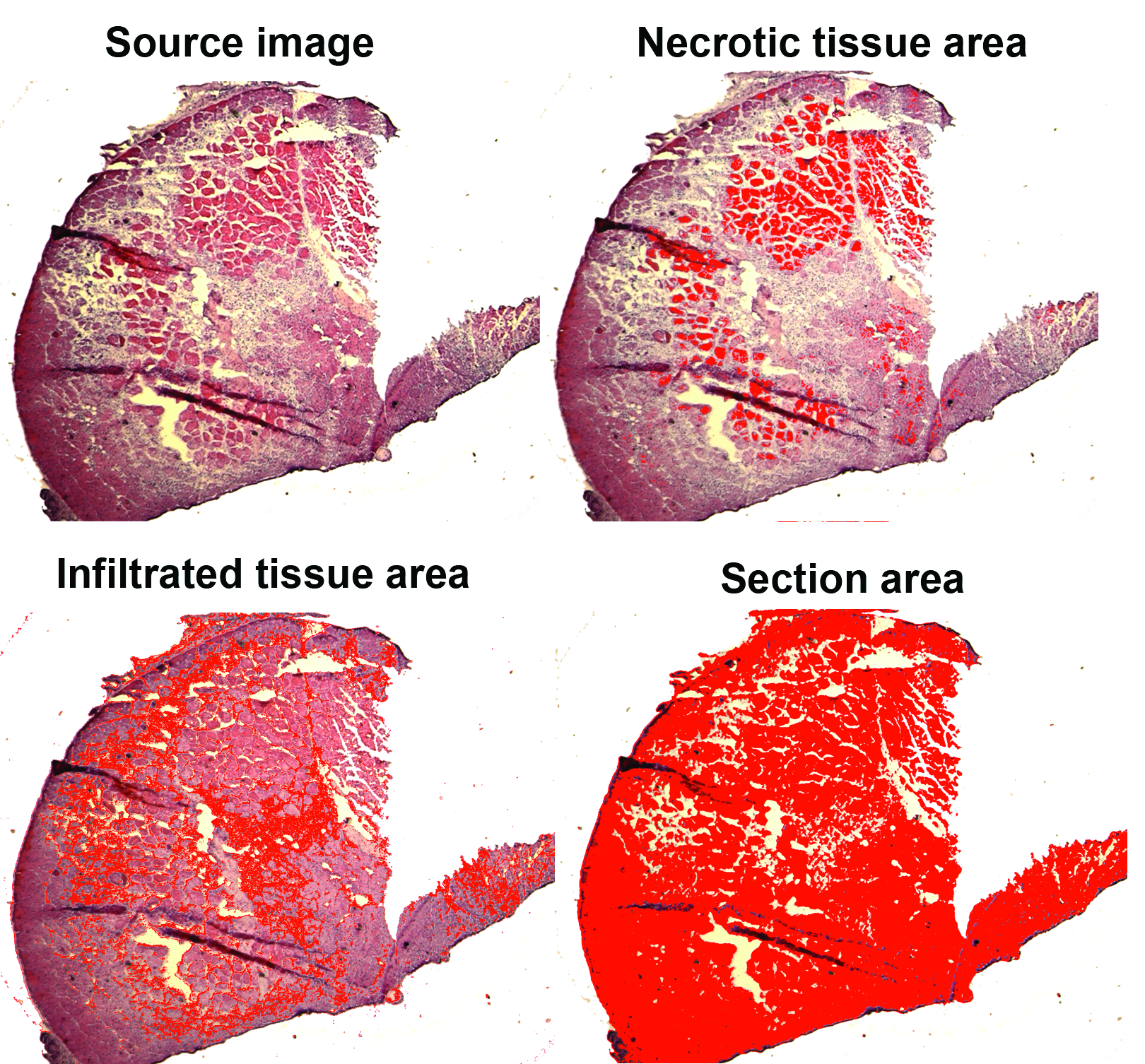

Supplement: Additional file 3: Figure S2. — Illustration for principle of morphometry adopted in tissue section studies. Highlighted regions (bright red) of necrotic and infiltrated tissue in a section of the tibial anterior muscle indicating color threshold selection of regions used for morphometry (hematoxylin/eosin staining). NIH ImageJ freeware for Mac used for processing. (TIFF 4432 kb) [file 13287_2015_199_MOESM3_ESM.tif]
